# Lack of Obvious Influence of PLLA Nanofibers on the Gene Expression of BMP-2 and VEGF during Growth and Differentiation of Human Mesenchymal Stem Cells

**DOI:** 10.1100/tsw.2009.36

**Published:** 2009-04-28

**Authors:** Markus D. Schofer, S. Fuchs-Winkelmann, C. Wack, M. Rudisile, R. Dersch, I. Leifeld, J. Wendorff, A. Greiner, J. R. J. Paletta, U. Boudriot

**Affiliations:** ^1^ Department of Orthopedics, University of Marburg, D-35043 Marburg, Germany; ^2^ Department of Chemistry, University of Marburg, D-35032 Marburg, Germany; ^3^ Department of Orthopedics, Sankt-Elisabeth-Hospital Gütersloh, D-33332 Güterslohoh, Germany

**Keywords:** nanofibers, tissue engineering, human mesenchymal stem cells, PLLA, BMP, VEGF

## Abstract

Growth factors like bone morphogenetic protein 2 (BMP-2) and vascular endothelial growth factor (VEGF) play an important role in bone remodeling and fracture repair. Therefore, with respect to tissue engineering, an artificial graft should have no negative impact on the expression of these factors. In this context, the aim of this study was to analyze the impact of poly(L-lactic acid) (PLLA) nanofibers on VEGF and BMP-2 gene expression during the time course of human mesenchymal stem cell (hMSC) differentiation towards osteoblasts. PLLA matrices were seeded with hMSCs and cultivated over a period of 22 days under growth and osteoinductive conditions, and analyzed during the course of culture, with respect to gene expression of VEGF and BMP-2. Furthermore, BMP-2–enwoven PLLA nanofibers were used in order to elucidate whether initial down-regulation of growth factor expression could be compensated. Although there was a great interpatient variability with respect to the expression of VEGF and BMP-2, PLLA nanofibers tend to result in a down-regulation in BMP-2 expression during the early phase of cultivation. This effect was diminished in the case of VEGF gene expression. The initial down-regulation was overcome when BMP-2 was directly incorporated into the PLLA nanofibers by electrospinning. Furthermore, the incorporation of BMP-2 into the PLLA nanofibers resulted in an increase in VEGF gene expression. Summarized, the results indicate that the PLLA nanofibers have little effect on growth factor production. An enhancement in gene expression of BMP-2 and VEGF can be achieved by an incorporation of BMP-2 into the PLLA nanofibers.

### INTRODUCTION

The reconstruction of large bony defects after injury or tumor resection often requires the use of graft material. Besides autologous bone grafts, artificial scaffolds based on synthetic biomaterials such as metals, ceramics, polymers, and composites have been developed[1,2]. Among them, scaffolds based on nanofibers are becoming more and more important[3,4]. These nanofibers can be produced by a broad spectrum of polymers, including biocompatible and biodegradable polymers, such as poly(glycolic acid) (PGA), poly(L-lactic acid) (PLLA), poly(ε caprolactone) (PCL), polyurethanes, polyphosphazenes, collagen, gelatin, and chitosan, as well as copolymers from the corresponding monomers in various compositions[5,6]. This results in the production of a broad spectrum of nanofiber-based scaffolds with different mechanical and biophysical properties. These fibers allow the differentiation of human mesenchymal stem cells (hMSCs) towards osteoblasts in principal[7,8,9]. Furthermore, they can be supplemented with growth factors like bone morphogenetic protein (BMP) or vascular endothelial growth factor (VEGF) in order to enhance osteoinductivity or angiogenesis.

BMP-2 is a well-studied member of the transforming growth factor beta (TGFβ) family known to induce osteoblast differentiation[10,11]. Meanwhile, this growth factor is used clinically to induce new bone formation in spine fusions and long bone nonunion fractures[12,13]. VEGF, a main regulator of angiogenesis[14], plays a crucial role in fracture repair[15] by attracting endothelial cells and by stimulating osteoblast differentiation[16]. Due to the fact that both growth factors play an important role in bone biology, this study was designed to analyze the impact of PLLA nanofibers on the expression of BMP-2 and VEGF.

### MATERIAL AND METHODS

#### Construction of Nanofibers and Characterization

The preparation of PLLA nanofibers and the incorporation of BMP-2 into the nanofibers, as well as their characterization by scanning electron microscopy (SEM) and analysis of static contact angles of water, has been reported earlier[17,18,19,20].

#### hMSC Isolation, Characterization, and Culture hMSCs were obtained from consenting patients with the approval of the institutional review board. The indication for surgery was primary osteoarthritis of the hip with increasing pain, decreased range of motion, and signs of progressive osteoarthritis in radiographs. The patients had no evidence of other bone or autoimmune diseases. The routinely removed bone was obtained from the proximal femur, while preparing the implant bed. MSCs were isolated and cultured according to the preparation of Pittenger et al.[21], with minor modifications as mentioned by Brendel et al.[22]. Each experiment was carried out four times, as described earlier[17,18,19,20].

#### Gene Expression Analysis

Extraction of RNA, synthesis of cDNA, as well as quantitative polymerase chain reaction (qPCR) analysis were performed as described earlier[17,18,19,20]. Primers (purchased at TIB Biomol, Berlin, Germany), cycle temperatures, and incubation times for VEGF[23], BMP-2, and 18s rRNA[24,25,26] were previously detailed. Additionally, VEGF-PCR products were analyzed by gel electrophoresis using 2% agarose.

### BMP-2 ELISA

BMP-2 was analyzed using ELISA kit Quantikine (R&D systems inc., Minneapolis, MN) according to the manufacturer.

### Statistics

All values were expressed as mean ± standard error of different patients as indicated in the text. Normal distribution was analyzed using the Kolmogorov-Smirnov-Test. Significances between the two groups were calculated using Students' t-test in the case of normal distribution. Otherwise, the Mann-Whitney U test was performed. Values of $p < 0.05$ were considered to be significant and marked with *. All calculations were done using the statistical analysis software SPSS 17.0.

## RESULTS

### Gene Expression of VEGF

In order to appraise the impact of PLLA nanofibers on angiogenesis, we first analyzed the ability of hMSCs to express VEGF. As shown in Fig. 1D, VEGF was expressed under both growth and osteoinductive conditions. The expression was limited to the occurrence of the splice variant 121. Furthermore, there was a time-dependent (but not significant) increase in the gene expression level of VEGF 121 in response to dexamethasone (Fig. 1A). The presence of PLLA nanofibers had no significant effect, either on the expression level of VEGF or on the occurrence of splice variants (Figs. 1B,C).

### Gene Expression of BMP-2

With respect to the expression of BMP-2, a different pattern could be observed. As shown in Fig. 2A, the presence of dexamethasone resulted in an early increase of BMP-2 gene expression. The presence of PLLA nanofibers resulted in a decrease of BMP-2 expression irrespective of the culture conditions (Figs. 2B–D).

### Influence of BMP-2 Incorporation on the Expression of Growth Factors

In order to elucidate whether the incorporation of BMP-2 is sufficient to compensate the decrease in gene expression of growth factors, we analyzed the time-dependent gene expression of VEGF and BMP-2 of hMSCs grown on PLLA-BMP-2 nanofibers and compared it to that obtained of hMSCs cultured on PLLA nanofibers alone. With respect to BMP-2 gene expression, an initial increase was observed when cells were cultured on PLLA-BMP-2 fibers, which normalized during the course of cultivation (Figs. 3A,B), regardless of whether growth or osteoinductive conditions were applied. Similar effects on VEGF expression could only be observed when cells were cultured under osteoinductive conditions, but not under growth conditions (Figs. 3A,B).

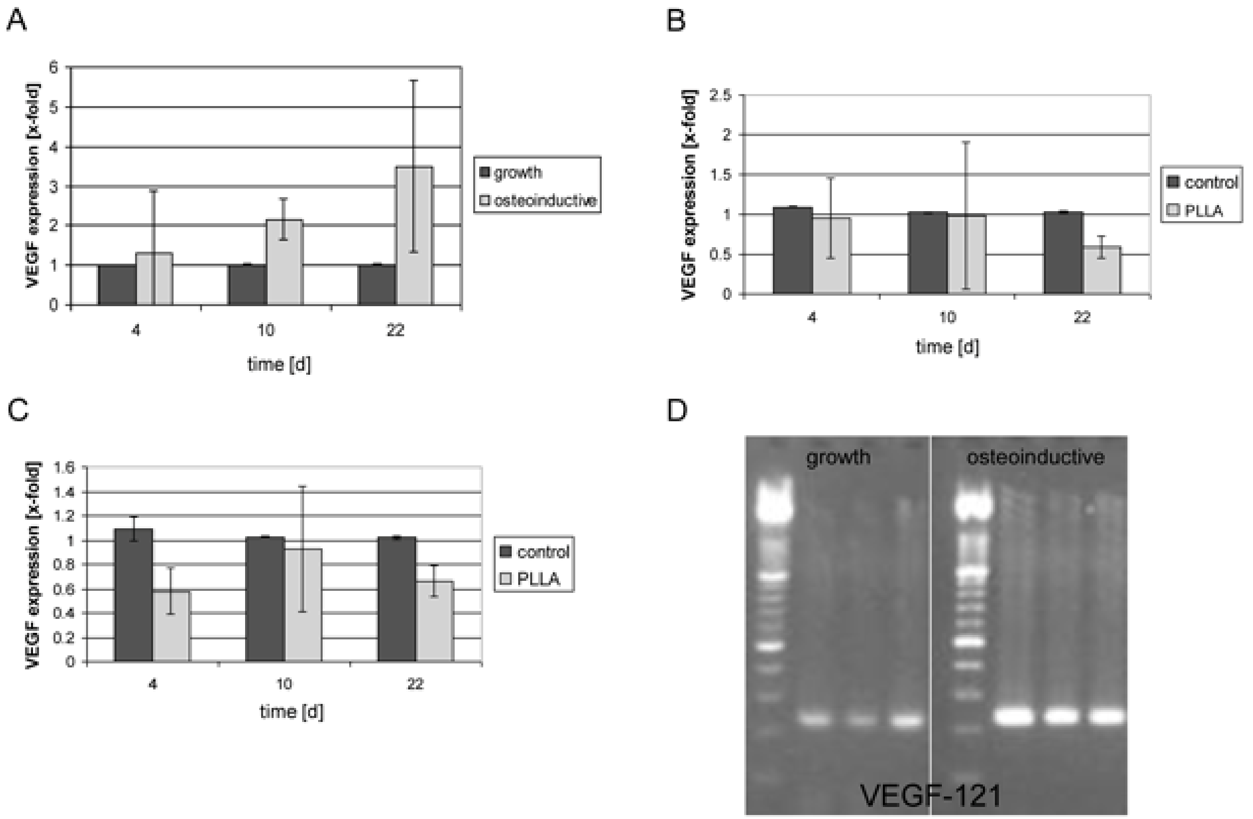

**FIGURE 1.** Influence of PLLA nanofibers on VEGF gene expression. Influence of dexamethasone on VEGF expression of hMSCs cultured on cover slips (A,D). Influence of PLLA nanofibers on VEGF expression of hMSCs cultured under osteoinductive (B) and growth conditions (C). (A–C) qPCR analysis normalized to 18S rRNA expression; error bars = standard error; n (number of replicates) = 4. (D) Agarose gel electrophoresis.

### DISCUSSION

PLLA is a biocompatible, biodegradable, and FDA-approved polymer that can easily be electrospun into a 3-D nonwoven network[27,28]. These constructs are appropriate as a matrix for osteoblast growth as well as for the osteogenic differentiation of hMSCs in principle[7,8,9]. However, there was a time-dependent down-regulation of genes, such as alkaline phosphatase, osteocalcin, and collagen-I, associated with the osteoblast lineage[19,20], indicating that the presence of PLLA nanofibers might influence the gene expression of other genes. If the expression of growth factors is affected, this might have an impact on implant healing.

Therefore, we analyzed the impact of PLLA nanofibers on the time-dependent expression of the BMP-2 gene during the differentiation of hMSCs. Although there was a great interpatient variability, our results indicate that BMP-2 is mainly expressed in the early stage of differentiation. While the effect of dexamethasone on BMP-2 expression of hMSCs is controversially discussed, our results confirmed findings by others, demonstrating that dexamethasone is involved in early BMP-2 up-regulation during hMSC differentiation[29]. If at all, PLLA nanofibers tend to result in a down-regulation in BMP-2 expression during the early phase of cultivation. This is supported by a down-regulation of genes associated with osteoblast differentiation as described earlier[19,20]. One explanation might be that, at least in part, an endocrine mechanism could be involved in the development of osteoblasts. This hypothesis is supported by the finding that supplementation of the PLLA nanofibers with BMP-2 compensates the down-regulation of BMP-2 and other genes of the osteoblast lineage[20].

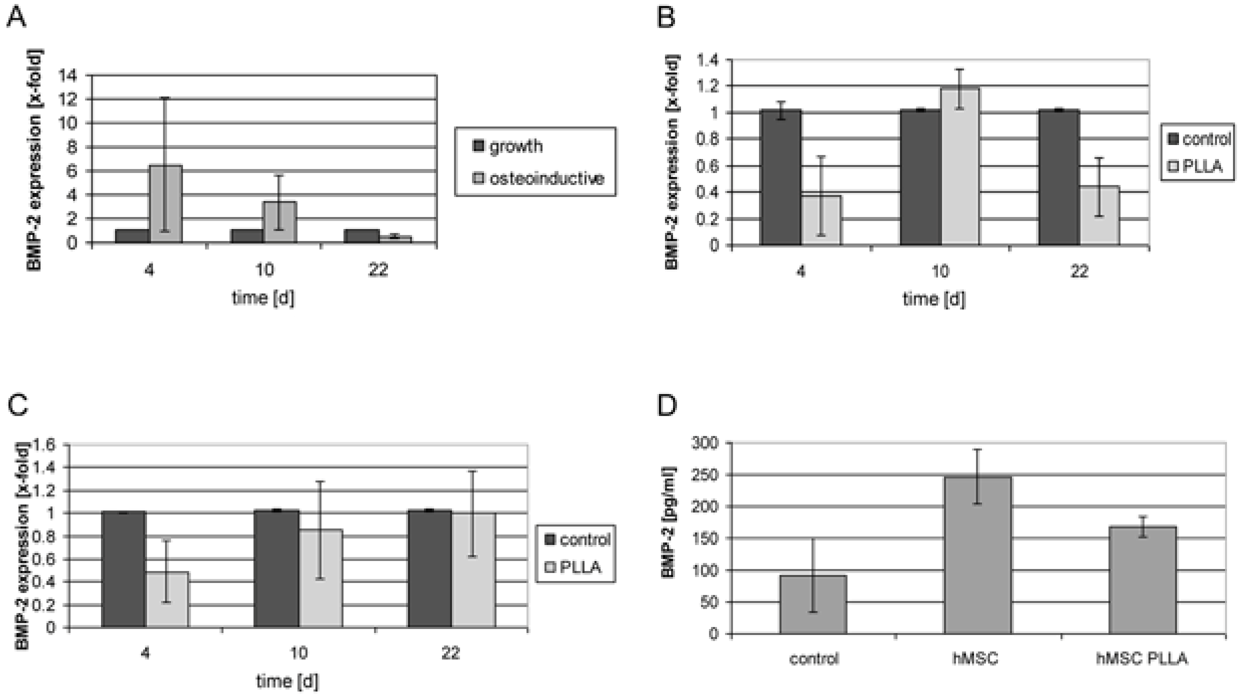

**FIGURE 2.** Influence of PLLA nanofibers on BMP-2 gene expression. Influence of dexamethasone on BMP-2 expression of hMSCs cultured on cover slips (A). Influence of PLLA nanofibers on BMP-2 expression of hMSCs cultured under osteoinductive (B) and growth conditions (C). BMP-2 protein concentration after 4 days of cultivation (D). (A–C) qPCR analysis normalized to 18S rRNA expression; error bars = standard error; n (number of replicates) = 4. (D) ELISA typical.

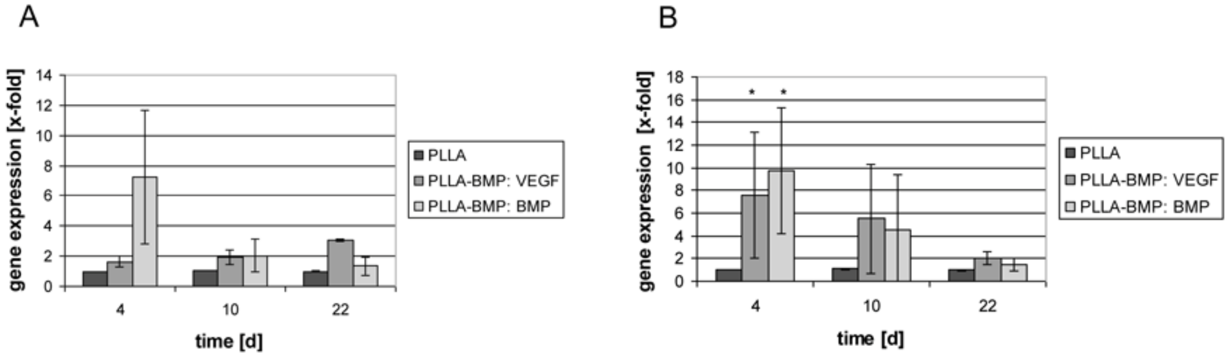

**FIGURE 3.** Influence of BMP-enwoven PLLA nanofibers on gene expression of VEGF and BMP-2. Time course of gene expression of hMSCs cultured under growth (A) and osteoinductive conditions (B), on BMP-enwoven PLLA nanofibers compared to PLLA nanofibers. (A–B) qPCR analysis normalized to 18S rRNA expression; error bars = standard error; n (number of replicates) = 4.

Due to the fact that VEGF-driven angiogenesis is a crucial process in fracture repair and wound healing[15,30], we analyzed the expression of this growth factor in order to appraise whether a supplementation of VEGF might be necessary when PLLA nanofiber constructs were used *in vivo*. Corresponding with the findings of other groups, VEGF 121 expression was stimulated by dexamethasone treatment along with osteoblastogenesis[31]. However, in contrast to the expression of BMP-2, we found no obvious influence of PLLA nanofibers on the expression of the VEGF gene. This suggests that PLLA nanofiber constructs will not compromise angiogenesis when used *in vivo*.

Furthermore, the effect that BMP-2 stimulated VEGF gene expression in osteoblasts[32] could also be observed when BMP-2 was incorporated into the PLLA nanofibers. Therefore, an incorporation of BMP-2 might suffice to trigger angiogenesis during bone healing *in vivo*.

### ACKNOWLEDGMENTS

This work was supported by the Deutsche Forschungsgemeinschaft (German Research Foundation, Grant No. BO 3065/1-1).
